# Authors’ Reply: The Transtheoretical Model: Is It Still the Best We Have?

**DOI:** 10.2196/79131

**Published:** 2025-07-07

**Authors:** Pernille Lunde, Asta Bye, Jostein Grimsmo, Are Hugo Pripp, Vibeke Ritschel, Even Jarstad, Birgitta Blakstad Nilsson

**Affiliations:** 1 Department of Rehabilitation Science and Health Technology, Faculty of Health Sciences, OsloMet – Oslo Metropolitan University, PB 4, St. Olavs Plass, Oslo, 0130, Norway, 47 48063537; 2 Department of Nursing and Health Promotion, Faculty of Health Sciences, OsloMet – Oslo Metropolitan University, Oslo, Norway; 3 European Palliative Care Research Centre (PRC), Department of Oncology, Oslo University Hospital and Institute of Clinical Medicine, University of Oslo, Oslo, Norway; 4 Department of Cardiac and Pulmonary Rehabilitation, Lovisenberg Rehabiliation, Cathinka Guldberg´s Hospital, Jessheim, Norway; 5 Oslo Centre of Biostatistics and Epidemiology, Oslo University Hospital, Oslo, Norway; 6 Department of Cardiology and Exercise Physiology, Norwegian Sport Medicine Clinic (Nimi, part of Volvat), Oslo, Norway; 7 Section for Physiotherapy, Division of Medicine, Oslo University Hospital, Oslo, Norway

**Keywords:** mHealth, cardiac rehabilitation, mobile phone app, smartphone, lifestyle, mobile health intervention, behavior change, chronic disease management, mobile health

We appreciate the thoughtful commentary and constructive perspectives by Cen and Lin [1] on our study, “Effects of Individualized Follow-Up With an App Postcardiac Rehabilitation: Five-Year Follow-Up of a Randomized Controlled Trial” [2]. This response aims to clarify aspects of our study that may have been misunderstood based on the claims presented by the authors [1].

First, Cen and Lin suggest that the most telling result is that the decline in outcomes began as early as 1 year. We would like to clarify that the exact timing of the decline is unknown. In our 2020 publication [3], we demonstrated that individualized follow-up via a mobile app was effective compared to usual care in improving peak oxygen uptake, exercise performance, exercise habits, and self-perceived goal achievement at the 1-year follow-up. At that point, the intervention ended, and both groups were transferred to usual care. Four years post intervention, with no contact with participants during this period, we observed that the previously demonstrated effects had diminished. Since no assessments were conducted between the 1-year and 5-year follow-ups, it remains unclear whether the decline began immediately after the intervention, at 1 year, or later.

Second, we appreciate that Cen and Lin raise the important issue of theoretical framework in intervention design. As emphasized by the Medical Research Council, the use of a theoretical framework and evidence-based strategies is essential for understanding mechanisms of change and evaluating complex interventions. While the intervention in our study was ongoing, we found the transtheoretical model appropriate as it acknowledges that behavior change is nonlinear, and the need for support and use of behavioral change techniques vary across stages [4]. However, Cen and Lin rightly question whether the model remains conceptually aligned when the intended outcome lies beyond the duration of the intervention. At the time of publishing our 1-year results, no prior study had demonstrated the effect of a 1-year app-based follow-up. This motivated us to seek funding for long-term follow-up. Had the 4-year results shown sustained effects, the findings would have been groundbreaking in addressing nonadherence in this population. However, as our recent results demonstrated, achieving the termination phase of behavior change within 1-year of follow-up appears unlikely for patients with cardiac disease. As noted in our paper [2], and in line with Cohen’s definition of adherence in cardiovascular risk reduction [5], long-term adherence depends on an ongoing, collaborative relationship between the patient and health care provider. Thus, if the goal is sustained self-management, the transtheoretical model may not be the most appropriate framework.
